# Association of serum leptin and ghrelin levels with smoking status on body weight: a systematic review and meta-analysis

**DOI:** 10.3389/fpsyt.2023.1296764

**Published:** 2023-12-04

**Authors:** Nour Shaheen, Ahmed Shaheen, Rehab Adel Diab, Abdelrahman M. Saad, Omar Ahmed Abdelwahab, Sama Soliman, Mahmoud Tarek Hefnawy, Alaa Ramadan, Mostafa Meshref, Abdulqadir J. Nashwan

**Affiliations:** ^1^Alexandria Faculty of Medicine, Alexandria University, Alexandria, Egypt; ^2^Faculty of Medicine, Al-Azhar University, Medical Research Group of Egypt, Cairo, Egypt; ^3^Faculty of Medicine, The Pavlov First State Medical University of St. Petersburg, St. Petersburg, Russia; ^4^Faculty of Medicine, Zagazig University, Medical Research Group of Egypt, Cairo, Egypt; ^5^Faculty of Medicine, South Valley University, Qena, Egypt; ^6^Neurology Department, Faculty of Medicine, Al-Azhar University, Cairo, Egypt; ^7^Nursing Department, Hamad Medical Corporation, Doha, Qatar

**Keywords:** smoking, leptin, ghrelin, obesity, cigarette smoking

## Abstract

**Background and aims:**

Smoking cigarettes is a major global health problem that affects appetite and weight. The aim of this systematic review was to determine how smoking affected plasma leptin and ghrelin levels.

**Methods:**

A comprehensive search of PubMed, Scopus, Web of Science, and Ovid was conducted using a well-established methodology to gather all related publications.

**Results:**

A total of 40 studies were included in the analysis of 11,336 patients. The overall effect showed a with a mean difference (MD) of −1.92[95%CI; −2.63: −1.20] and *p* = 0.00001. Subgroup analysis by study design revealed significant differences as well, but with high heterogeneity within the subgroups (*I*^2^ of 82.3%). Subgroup by sex showed that there was a significant difference in mean difference between the smoking and non-smoking groups for males (MD = −5.75[95% CI; −8.73: −2.77], *p* = 0.0002) but not for females (MD = −3.04[95% CI; −6.6:0.54], *p* = 0.10). Healthy, pregnant, diabetic and CVD subgroups found significant differences in the healthy (MD = −1.74[95% CI; −03.13: −0.35], *p* = 0.01) and diabetic (MD = −7.69[95% CI, −1.64: −0.73], *p* = 0.03). subgroups, but not in the pregnant or cardiovascular disease subgroups. On the other hand, the meta-analysis found no statistically significant difference in Ghrelin serum concentration between smokers and non-smokers (MD = 0.52[95% CI, −0.60:1.63], *p* = 0.36) and observed heterogeneity in the studies (*I*^2^ = 68%).

**Conclusion:**

This study demonstrates a correlation between smoking and serum leptin/ghrelin levels, which explains smoking’s effect on body weight.

**Systematic review registration:**

https://www.crd.york.ac.uk/ prospero/display_record.php, identifier (Record ID=326680).

## Introduction

Smoking is a major global health problem that has been associated with various metabolic and neurologic effects on appetite (1, 2). Smoking has been linked to the development of central obesity, resistance to insulin, low levels of high-density lipoprotein, and type 2 diabetes (3, 4). In the short term, the nicotine found in cigarettes can increase energy expenditure and decrease appetite, which may explain why smokers generally have lower body weight than non-smokers. However, when smokers quit, they often experience weight gain. On the other hand, individuals who smoke heavily tend to have higher body weights than light smokers or non-smokers (5). Leptin is a hormone primarily secreted by adipocytes in white adipose tissue and helps regulate energy balance by decreasing hunger (6). Leptin controls hunger and aids in maintaining a healthy weight. When it functions properly, it regulates the balance between the amount of food consumed and the amount of fat stored in the body. High levels of leptin signal to the brain that the fat cells are saturated, thereby reducing hunger (7). However, the relationship between smoking and serum leptin is still debated. Some studies have found that smoking has been linked to increased leptin levels, while smoking cessation has been associated with decreased leptin levels (8–10) and some other studies showed the reverse (11, 12). Ghrelin, another hormone primarily secreted by the stomach, regulates energy balance, metabolism, and gastrointestinal function. Ghrelin stimulates hunger and promotes lipogenesis (13, 14). Studies have shown that smoking cigarettes can increase ghrelin levels in the blood, and smoking cessation causes it to decrease (15–17).

To date, no systematic review or meta-analysis has attempted to assess the effect of smoking on plasma leptin and ghrelin concentrations. The purpose of this study is to determine the effect of cigarette smoking on plasma levels of leptin and ghrelin, thus adding a cornerstone to the evidence about understanding the effect of smoking cigarettes on body weight.

## Methods

This systematic review and meta-analysis were conducted in accordance with the Cochrane Handbook for Systematic Reviews of Interventions (18) and the Preferred Reporting Items for Systematic Reviews and Meta-Analyses (PRISMA) guidelines (19). The study protocol was registered in PROSPERO (CRD42022326680), and any deviations from the protocol were reported in the Methods section.

### Eligibility criteria

#### Inclusion criteria

- Original articles that report on the effect of smoking on plasma leptin and ghrelin levels.
- Clinical studies, including randomized controlled trials and observational studies such as cohort and case-control studies.
- Adult subjects (18 years and above) were included in the studies.
- Studies that used nicotine/smoking as the intervention and measured serum ghrelin and leptin levels as the outcome measures.

#### Exclusion criteria

- Studies with unclear data or missing information.
- Studies that used other food supplements containing nicotine as the intervention.
- Short-term studies (duration of less than 1 week) were excluded from the analysis.
- Studies did not report the effect of smoking on plasma leptin and ghrelin levels.
- Studies that did not report the data necessary for the meta-analysis, such as means and standard deviations for ghrelin and leptin levels.

### Information sources and search strategy

We thoroughly searched four internet databases (PubMed, Scopus, Web of Science, and Ovid). The search strategy for each database is presented in (Supplementary material). The listed studies’ references were carefully examined for any possible eligible articles. The search was carried out by two independent reviewers (NS and AS).

### Selection process

Endnote (Clarivate Analytics, United States) was used to get rid of duplicates, and then the returned references went through two steps of screening:

*Step 1: title and abstract screening.*

The first step involved screening the titles and abstracts of all papers retrieved by the search strategy for relevance by two independent reviewers (RD and AS). Studies that did not meet the inclusion criteria or had any exclusion criteria were discarded.

*Step 2: full-text screening.*

The full texts of the remaining studies were then downloaded and reviewed in detail by two independent reviewers (RD and AS). These reviewers evaluated the eligibility of the studies based on the predefined inclusion and exclusion criteria. Any disagreements on the eligibility of a particular study were resolved through discussion between the reviewers or by a third reviewer (NS).

### Data extraction

After the eligibility of the studies was determined, the reviewers extracted relevant data from the included studies. This data included information such as study design, sample size, population characteristics, and the main outcome measures. Three reviewers independently performed data extraction (RD, SS, and AM), and any discrepancies were resolved through discussion or by a third reviewer (NS).

### Quality assessment of the included studies

The risk of bias of the included studies in the meta-analysis was assessed using the Newcastle-Ottawa Scale (NOS) (20) by two independent authors (MH and SS). The NOS is a tool commonly used to assess the quality of observational studies. The guidelines for using the NOS were followed for each study design, including case-control, cohort, and cross-sectional studies, without any modifications to the scale. The NOS evaluates the risk of bias in three domains: selection, comparability, and exposure/outcome assessment.

For each domain, the studies were given a certain number of stars based on the level of quality. The final scores were then converted to The Agency for Healthcare Research and Quality (AHRQ) standards of good, fair, and poor quality. One or two stars in the comparability domain, three or four stars in the selection domain, and two or three stars in the exposure/outcome domain were given to studies to indicate their high quality. If a study earned two stars for selection, one or two stars for comparability, and two or three stars for exposure/outcome, it was deemed to be of fair quality. No or one star in the domain of selection, the domain of comparability, and the area of exposure/outcome indicated a low-quality study (20).

### Data analysis

The meta-analysis utilized RevMan 5.4 for calculation of weighted mean differences (WMDs) and standard errors (SEs) in serum ghrelin and leptin levels. WMDs, representing the difference in mean hormone levels between smokers and non-smokers, were weighted by the inverse of variance. SEs were computed as the square root of within-study and between-study variance sums. Random-effects model, accommodating heterogeneous studies, assumed a normal distribution of true effects with a mean and variance. Mean differences (MD) as the effect size were calculated for uniformity. Heterogeneity was assessed via chi-square test and *I*-squared statistic, considering significance at *p* < 0.05 and *I*^2^ > 50%. Funnel plots and Egger’s regression/Begg and Mazumdar tests were used for publication bias analysis. Subgroup analysis explored heterogeneity sources, categorizing studies by design, gender, health status, and pregnancy. Significance was set at *p* < 0.05 for chi-square tests in subgroup analysis. The studies with small numbers of effects did not show different treatment effects than large ones even after performing Hartung-Knapp adjustment (21).

## Results

### Literature search results

**Figure 1:**
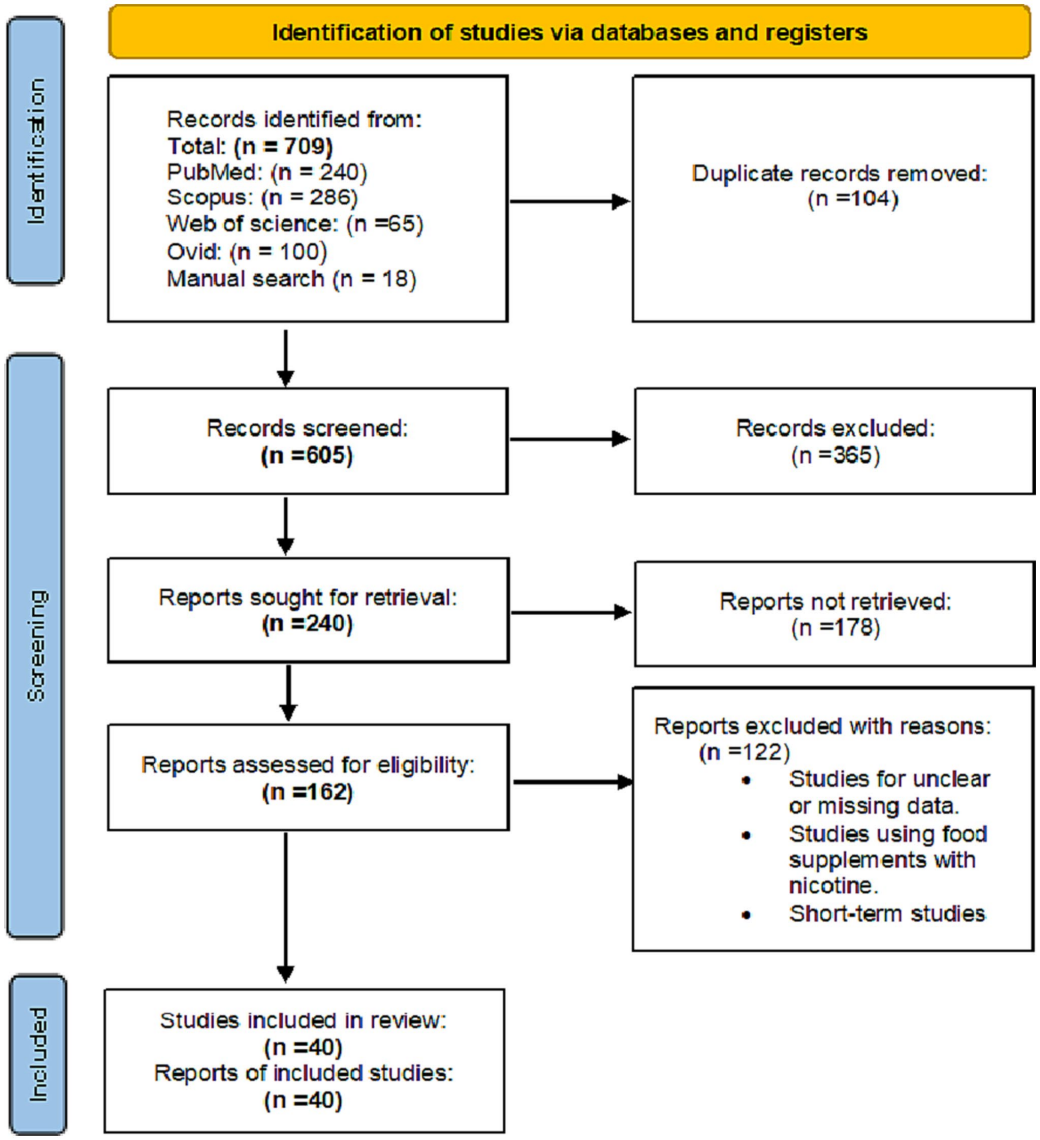
PRISMA flow diagram of studies’ screening and selection.

A total of 709 records were identified through searching the PubMed, Google Scholar, Scopus, Web of Science and Ovid databases. After removing duplicates, 365 records were excluded for being unrelated to the study topic. A total of 240 records were screened for relevance, and 178 were excluded after the full-text review. A total of 162 full-text articles were assessed for eligibility, and 40 studies were included in the qualitative and quantitative (meta-analysis) synthesis, with 40 studies being included in the meta-analysis (Figure 1).

### Characteristics of the included studies

This meta-analysis included 40 studies: 18 case-control studies, 14 cross-sectional studies, and 8 cohort studies. The included studies have a pooled total of **11, 336** patients: **4,083** of whom were smokers with a mean age of 43.39 ± 0.46 years and 60% were males, while **7,253** were non-smokers with a mean age of 40 ± 0.47 and 49.7% were males. Most papers included healthy individuals except Goltz et al. (22) and Wei et al. (23) included patients with CVD risk factors, Bokarewa et al. (24) studied fibromyalgia patients, Aşçibaşi et al. (25) studied patients with psychiatric and chronic diseases and 4 papers [Mutairi et al. (26), Yoshinari et al. (27), Al-Daghri et al. (28), and Targher et al. (29)] included diabetic participants (Table 1). According to the NOS tool, eighteen of the included studies had good quality, seven had fair quality, and fifteen had poor quality. The overall bias is present in Table 1 and the details for each domain are present in (Supplementary material).

**Table 1:** Baseline characteristics and quality score of the included studies.

Study ID	Study design	Country	Overall Quality Score according to the NOS tool	Smoking group			Non-smoking group			Health status
				No.	Age (y)	Sex: Male (M)/Female(F)	No.	Age(y)	Sex	
Hilawe et al. (30)	Cohort	Japan	Good	954	58.8 ± 17.044	M: 898, F:56	1,608	46.2 ± 7.1	M:933, F:675	Hypertension, diabetic and healthy
Goltz et al. (22)	Case-control	Germany	Good	60	33.95 ± 9.99	M:20, F:60	64	33.53 ± 9.28	M:24, F:40	Healthy
Kianoush et al. (31)	Cross-sectional	US	Poor	213	59.7 ± 8.6	M:120, F:93	871	64.3 ± 9.8	M:332, F:539	Healthy
Gomes et al. (32)	Cross-sectional	Brazil	Poor	24	25.31 ± 9.72	–	22	25.31 ± 9.72	–	Healthy
Bokarewa et al. (24)	Case-control	Sweden	Fair	18	51 ± 2.4	All females	19	51 ± 2.4	All females	Fibromyalgia
Ozkan et al. (33)	Case-control	Turkey	Good	21	26.8 ± 3.6	All females	23	26.6 ± 4	All females	Healthy
Lucas et al. (34)	Cross-sectional	Spain	Poor	293	39.9 ± 13.3	M:113, F:179	600	47.6 ± 15.1	M: 366, F:234	Healthy
Klein et al. (35)	Case-control	USA	Fair	19	22.2668 ± 3.8878	M:11, F:8	22	24.4527 ± 5.4799	M:11, F:10	Healthy
Perkins et al. (8)	Case-control	USA	Good	31	37.1 ± 6.6	–	21	33.8 ± 3.8	–	Healthy
Bergmann et al. (36)	Case-control	Germany	Poor	56	48.8 ± 48.8	All females	257	56.257 ± 11.07	All females	Healthy
Helland et al. (37)	Cohort	Norway	Good	152	28.3 ± 5.2	All females	266	28.3 ± 3.7	All females	Healthy
Nicklas et al. (38)	Case-control	USA	Good	22	62 ± 7.8	All males	22	64 ± 8	All males	Healthy
Wei et al. (23)	Cross-sectional	USA	Poor	20	46.04 ± 25.693	M:14, F:6	74	46.04 ± 25.693	F:60, M:24	CVD risk factors
Ekmekci et al. (39)	Case-control	Turkey	Poor	22	58.06 ± 11.07	–	40	58.06 ± 11.07	–	Healthy
Koc et al. (40)	Case-control	Turkey	Good	54	21.18 ± 11.9	All males	26	21.69 ± 15.2	All males	Healthy
Mutairi et al. (26)	Case-control	Kuwait	Good	76	44.0771 ± 6.32	All males	128	45.01 ± 5.389	All males	Healthy and diabetic
Coutant et al. (41)	Cross-sectional	France	Good	30	27.2 ± 5.2	All females	57	28.4 ± 4.4	All females	Healthy
Mantzoros et al. (42)	Cohort	USA	Good	48	34.7 ± 4.15	All females	125	34 ± 1.35	All females	PCOS and healthy Women
Hara et al. (43)	Cross-sectional	Japan	Poor	400	45.657 ± 6.4	All males	198	48.9 ± 7	All males	Healthy
Larsson et al. (44)	Cross-sectional	Sweden	Fair	26	58.6 ± 0.03	All females	75	58.6 ± 50.03	All females	They were healthy except for impaired glucose tolerance in 18 patients
Yoshinari et al. (27)	Cross-sectional	Japan	Fair	17	52.2 ± 6.9	All males	20	55 ± 4.9	All males	Type 2 Diabetes
Al-Daghri et al. (28)	Cross-sectional	KSA	Poor	55	44 ± 11.7	All males	70	51.6 ± 8.3	All males	Type 2 Diabetes
Targher et al. (29)	Cross-sectional	Italy	Poor	30	31.67 ± 1.281	–	44	31.645 ± 1.28	–	Type 1 diabetic and healthy
Pérez-Bautista et al. (45)	Case-control	Mexico	Good	140	40.195 ± 34.5419	All females	70	67.26 ± 7.32	All females	Women with COPD
Stadler et al. (46)	Cohort	USA	Fair	27	28.9 ± 6.2	M:18, F:9	14	27 ± 5.2	F: 5, M:9	Healthy
Bouhours-Nouet et al. (47)	Case-control	France	Good	42	28.1 ± 4.9	M: 14, F:28	42	28.2 ± 4.0	M:17, F:26	Healthy pregnant mothers and newborns
Kim et al. (48)	Case-control	Korea	Poor	198	37.2 ± 6.6	All males	117	40.1 ± 10.1	All males	Healthy
Lee et al. (15)	Cohort	Korea	Poor	28	37.2 ± 6.6	All males	18	37.2 ± 6.6	All males	Healthy
Kaabi et al. (49)	Cross-sectional	KSA	Poor	30	27	All males	30		All males	Healthy
Kryfti et al. (50)	Cohort	Greece	Good	35	56.1 ± 9.4	M:19, F: 16	26	56.1 ± 9.4	F: 22	Healthy
Machado et al. (51)	Case-control	Brazil	Fair	129	44.77 ± 11.02	M:36, F:93	85	40.14 ± 12.86	F:67	Healthy, CVS, DM, sexual abuse, emotional abuse, blood hypertension, and mood disorders
Rao et al. (52)	Case-control	India	Poor	64	28.3 ± 4.03	All females	121	26.11 ± 4.263	All females	Healthy
Aşçibaşi et al. (25)	Case-control	Turkey	Good	40	24.6 ± 3.1	M:26, F:14.	40	23.87 ± 2.57	M:26, F:14	Psychiatric disorder: 4, chronic disorder: 4
Bai et al. (53)	Case-control	China	Good	50	52 ± 11	All males	46	50 ± 12	All males	Healthy
Mutschler et al. (54)	Cohort	Switzerland	Poor	10	51.4 ± 13.5	All males	10	51.4 ± 13.5	All males	Healthy
Hussain et al. (55)	Case-control	KSA	Good	31	48.5 ± 9.3	All males	35	48.1 ± 6.1	All males	Healthy
Nagayasu et al. (56)	Cross-sectional	Japan	Good	93	4.6849 ± 8.549	All males	267	14.6 ± 7.4704	All males	Healthy
Al′Absi et al. (57)	Cross-sectional	USA	Poor	32	34.06 ± 4.67	F: 14, M: 18	32	34.1 ± 14.6	F: 14, M: 18	Healthy
Martin et al. (58)	Cohort	USA	Fair	447	40	–	1,597	40	–	Healthy
Cobanoglu et al. (59)	Cross-sectional	Cyprus	Good	46	8.4 ± 1.3	F: 27, M: 19	51	8.6 ± 1.6	F:25/M: 26	Healthy

### Serum leptin levels in smokers and non-smokers

#### Study designs-based subgroup analysis

**Figure 2:**
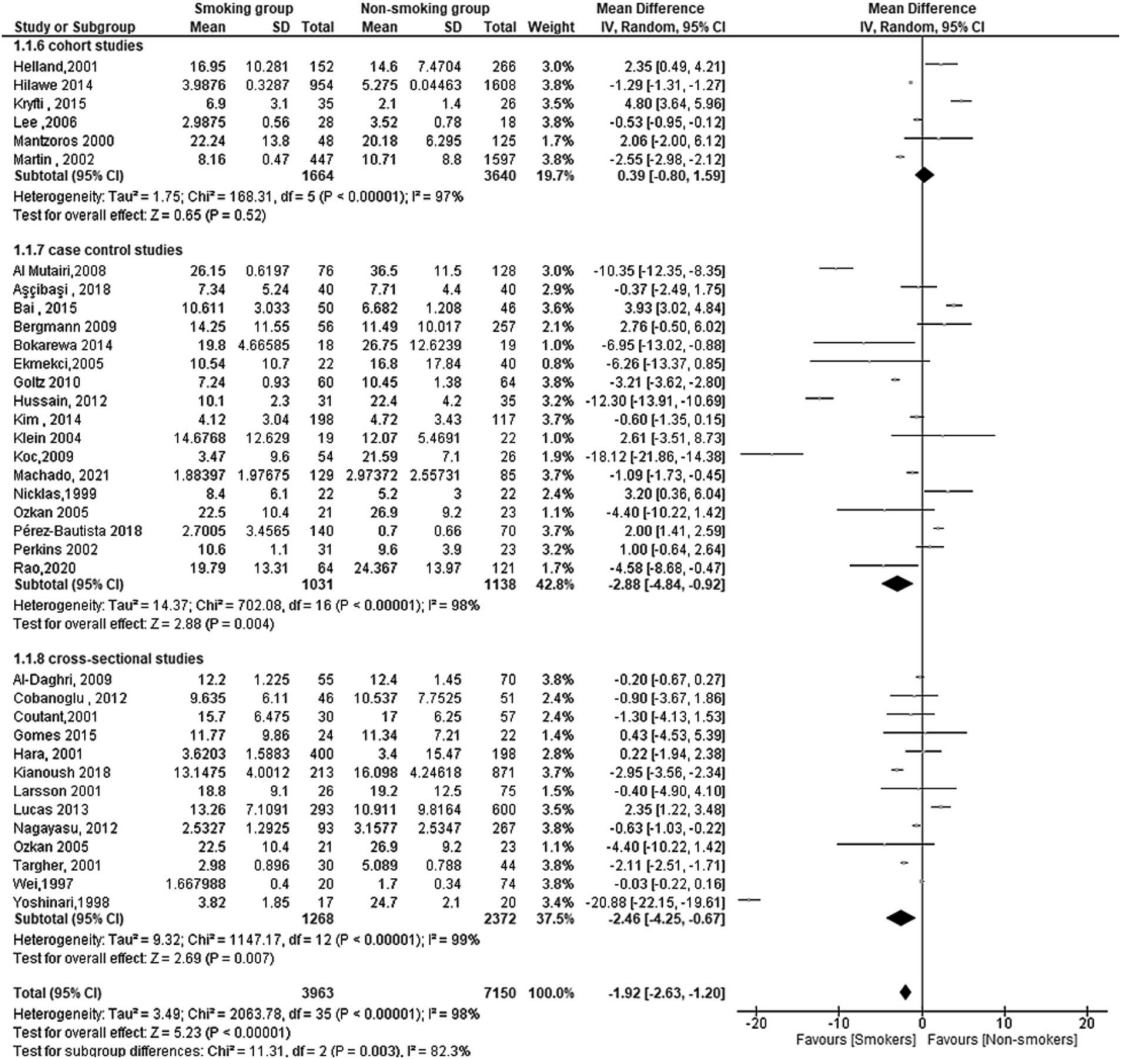
Forest plot diagram of serum leptin concentration between smokers and non-smokers according to the study designs.

A total of 35 studies reported serum leptin in smoking and non-smoking groups. The overall effect was significant, with a mean difference (MD) of −1.92[95%CI, −2.63:1.20] and *p*-value of 0.00001. Subgroup analysis by study design (cohort, case-control, and cross-sectional) revealed significant differences with a Chi^2^ of 11.31, df of 2, and a *p* value of 0.003. The heterogeneity within the subgroups was high, with an *I*^2^ of 82.3% (Figure 2).

For the cohort studies, the overall effect was not significant (MD = 0.39[95% CI; −0.80:1.59], *p* = 0.52) with high heterogeneity (Tau^2^ = 1.75, Chi^2^ = 168.31 df = 5 (*p* < 0.00001); *I*^2^ = 97%).

For the case-control studies, the overall effect was significant (MD = −2.88[95% CI; −4.84: −0.92], *p* = 0.004) with high heterogeneity (Tau^2^ = 14.37, Chi^2^ = 702.08, df = 16 (*p* < 0.00001); *I*^2^ = 98%).

Finally, for the cross-sectional studies, the overall effect was significant (MD = −2.46[95% CI; −4.25: −0.67], *p* = 0.007) with high heterogeneity (Tau^2^ = 9.32; Chi^2^ = 1147.17, df = 12 (*p* < 0.00001); *I*^2^ = 99%).

### Risk of bias results

**Figure 3:**
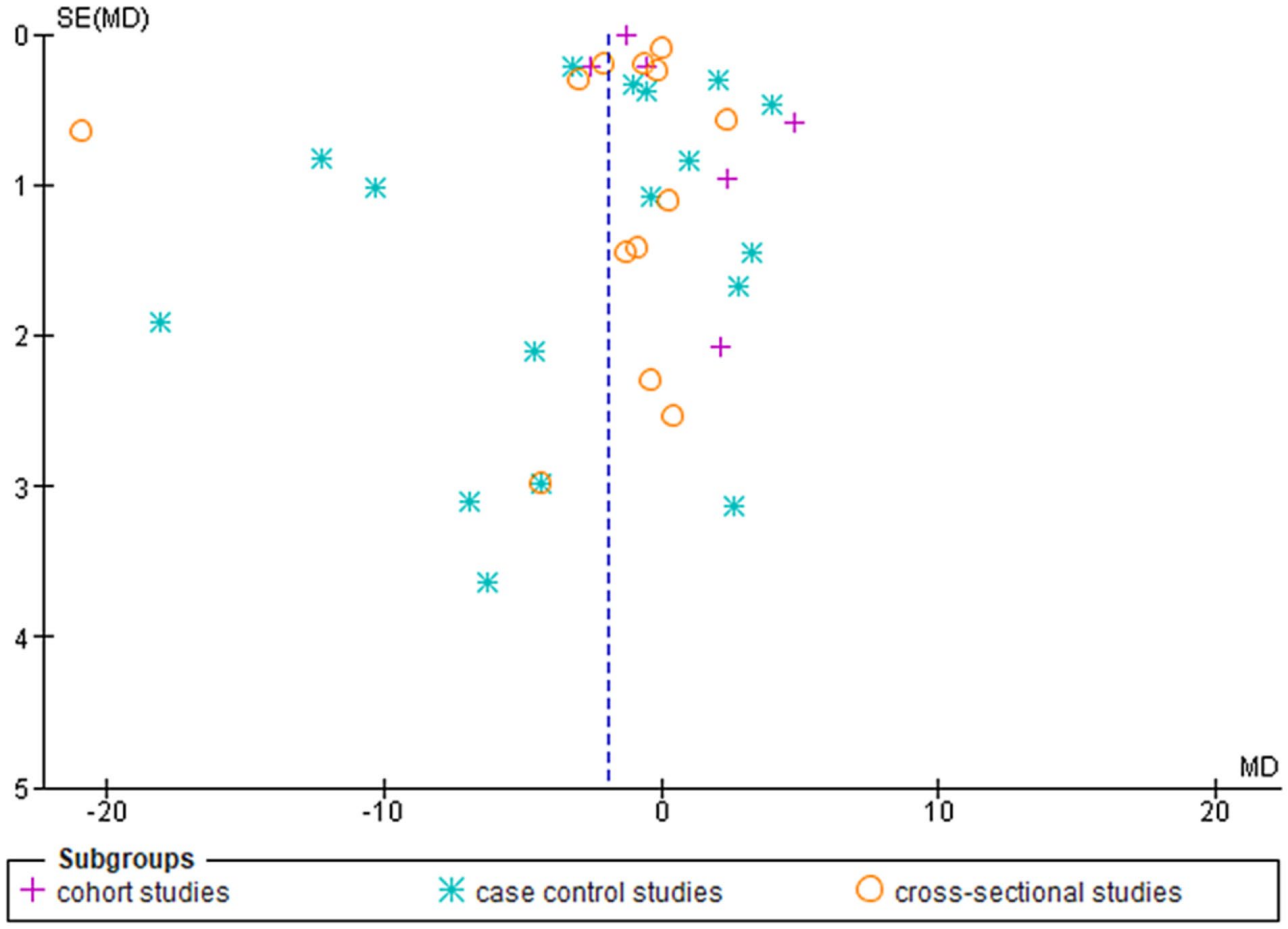
Shows the funnel plot investigating the publication bias among the studies in reporting serum leptin with subgrouping according to the study design.

In summary, this meta-analysis found a significant overall difference in serum leptin levels between smoking and non-smoking groups, as the smoking group had a lower level of leptin, with subgroup analysis revealing significant differences by study design. Moreover, the heterogeneity within the subgroups was high (Figure 3).

#### Sex-based subgroup analysis

The results of the meta-analysis show that there is a significant difference in mean difference between smoking and non-smoking groups. So, subgroup analysis was conducted to examine the effect of gender on this relationship.

For males, the test for overall effect (MD = −5.75[95% CI; −8.73: −2.77], *p* = 0.0002) showed a strong association between smoking and leptin levels, with lower levels of leptin in the smoking men group. Heterogeneity was also high among the studies included in this subgroup, as indicated by Tau^2^ = 22.43, Chi^2^ = 1421.21, df = 9 (*p* < 0.00001) and *I*^2^ = 99%.

**Figure 4:**
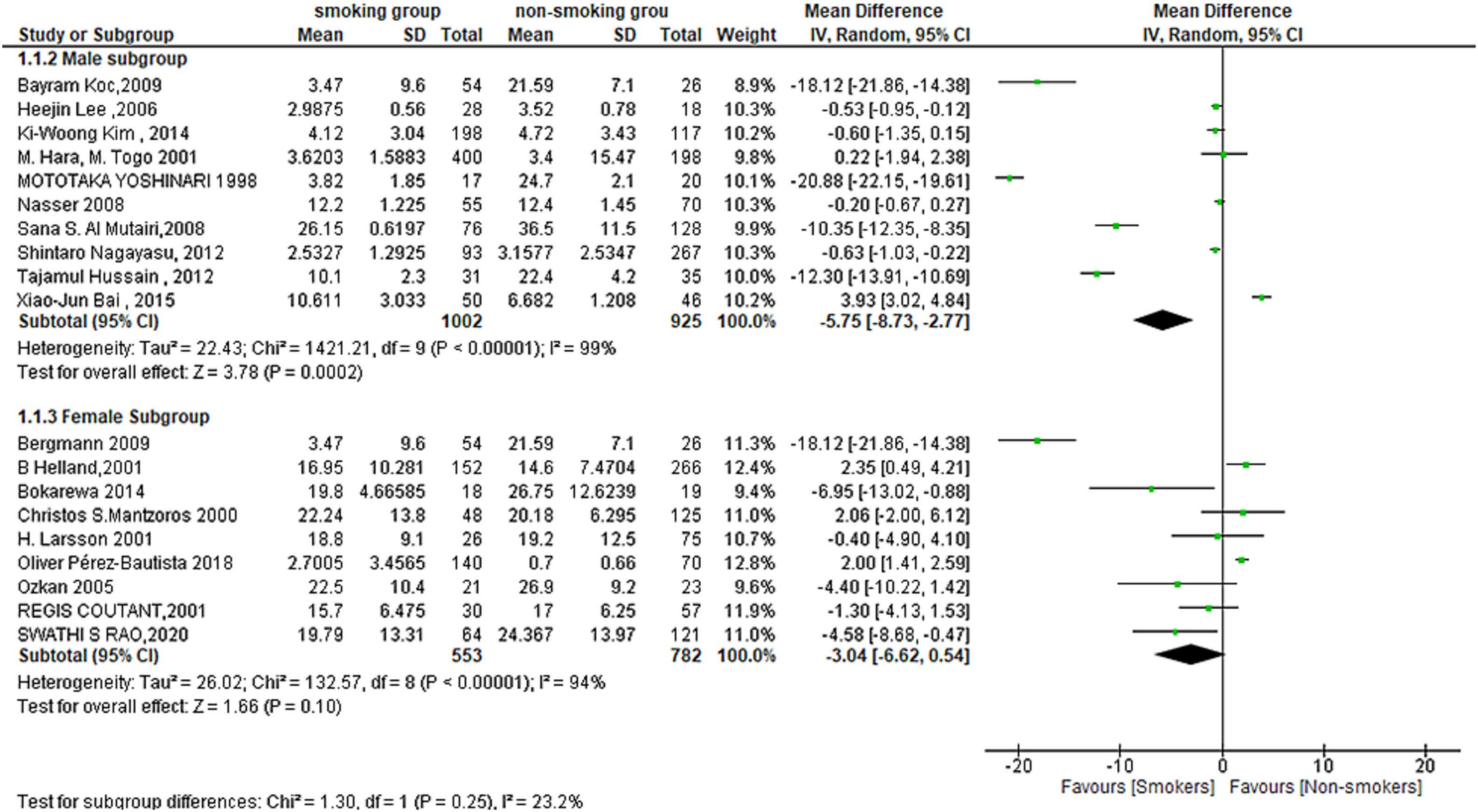
Forest plot diagram of serum leptin concentration between smokers and non-smokers according to the sex.

In contrast, the overall effect for Females was not statistically significant (MD = −3.04[95% CI; −6.6:0.54], *p* = 0.10). Heterogeneity was also high among the studies included in this subgroup, as indicated by Tau^2^ = 26.02, Chi^2^ = 132.57, df = 8 (*p* < 0.00001) and *I*^2^ = 94%. This subgroup showed also a lower level of leptin in the smoking women group despite the non-statistically significant difference (Figure 4).

**Figure 5:**
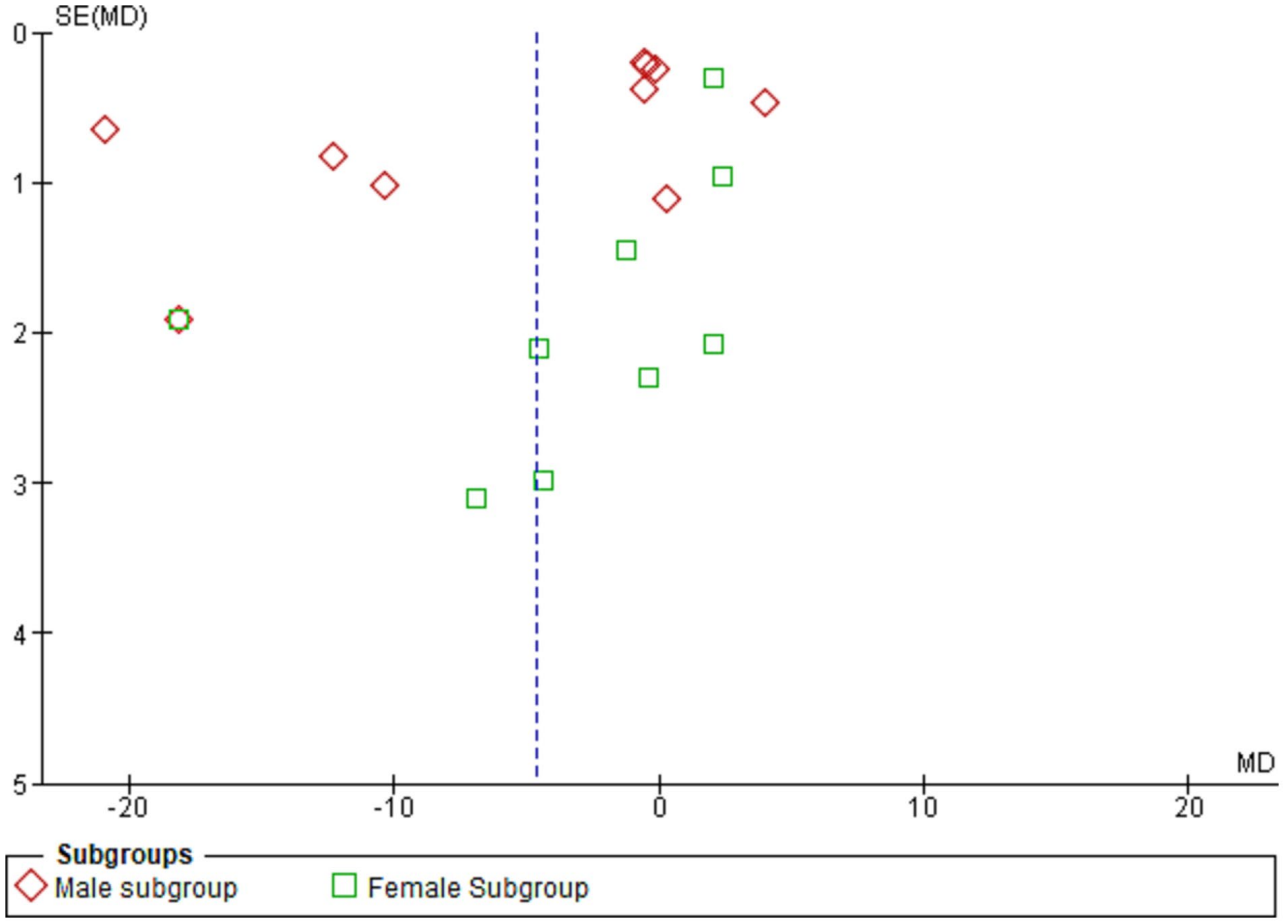
Shows the funnel plot investigating the publication bias among the studies in reporting serum leptin with subgrouping according to sex.

Figure 5 shows the funnel plot investigating the publication bias among the studies in reporting serum leptin with subgrouping according to sex.

### Other subgroups

**Figure 6:**
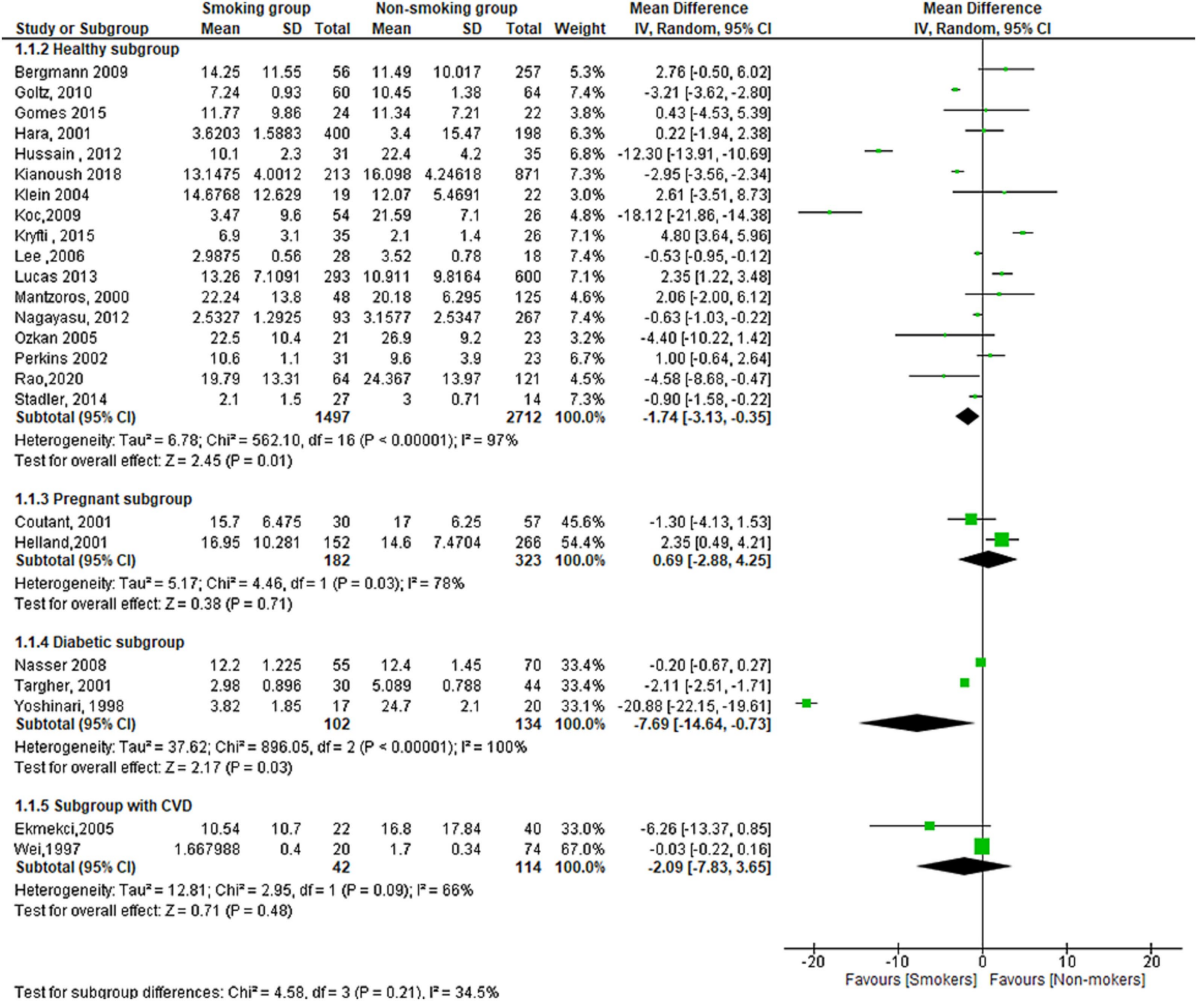
Forest plot diagram of serum leptin concentration between smokers and non-smokers according to the healthy status, pregnancy, diabetic, and CVD groups.

The results of subgroup analysis using (healthy, pregnant, diabetic and CVD subgroups) showed that there is a statistically significant difference in leptin serum concentration between smokers and non-smokers in the healthy subgroup (MD = −1.74[95% CI; −03.13: −0.35], *p* = 0.01) and diabetic subgroup (MD = −7.69[95% CI; −1.64: −0.73], *p* = 0.03). However, there was no statistically significant difference in the pregnant subgroup (MD = −0.69[95% CI; −2.88:4.25], *p* = 0.71) or the subgroup with cardiovascular disease (MD = −2.09[95% CI; −7.83: −3.65], *p* = 0.48). The meta-analysis also found high levels of heterogeneity in the healthy subgroup (*I*^2^ = 97%), diabetic subgroup (*I*^2^ = 100%), and subgroup with cardiovascular disease (*I*^2^ = 66%) (Figure 6).

**Figure 7:**
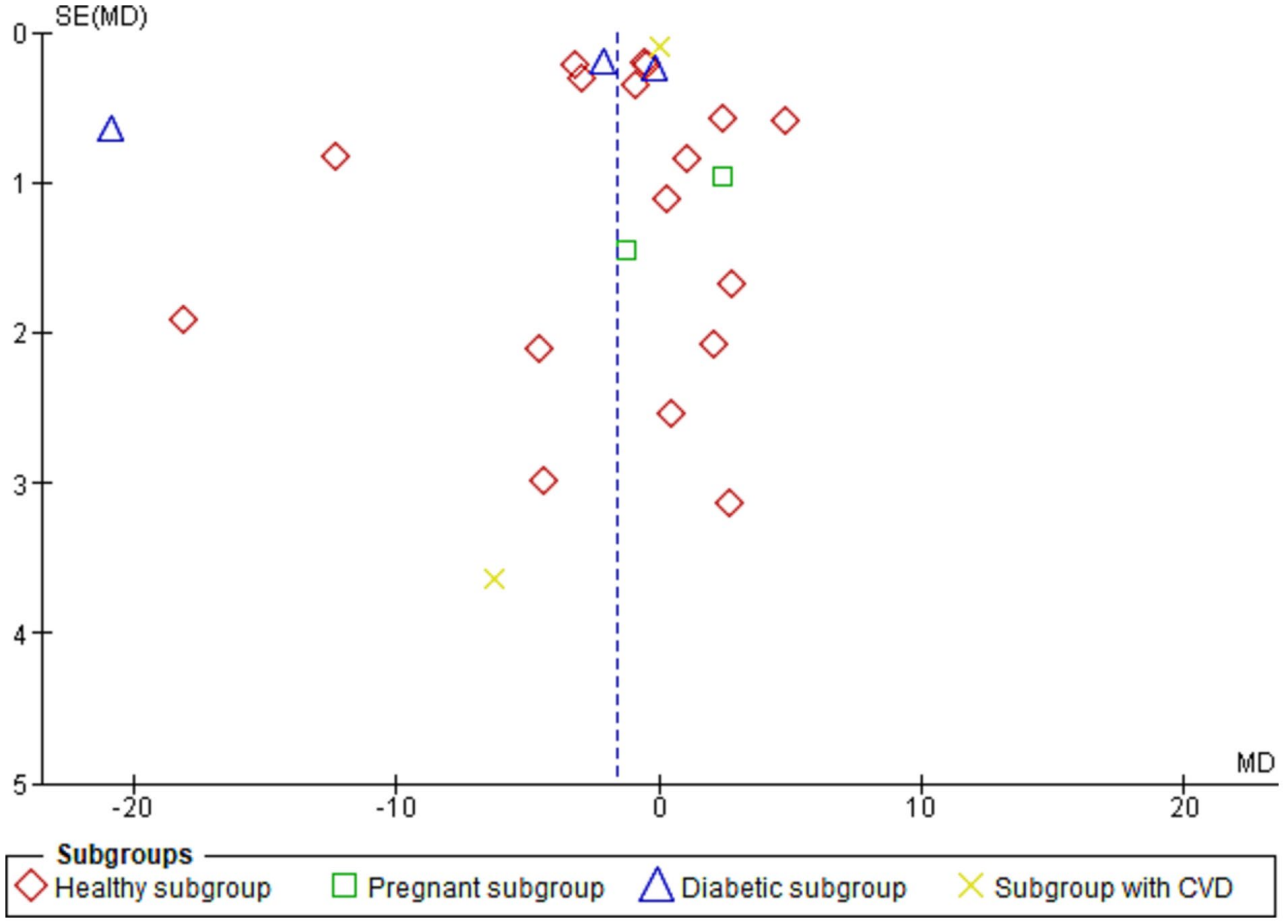
Shows the funnel plot investigating the publication bias among the studies in reporting serum leptin with subgrouping according to the health condition.

Figure 7 shows the funnel plot investigating the publication bias among the studies in reporting serum leptin with subgrouping according to the health condition.

### Serum ghrelin levels in smokers and non-smokers

**Figure 8:**
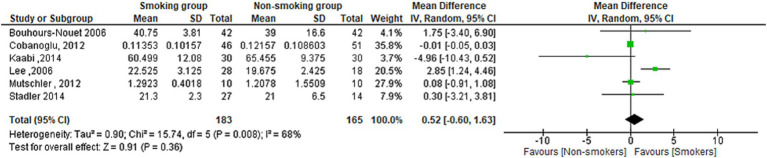
Forest plot diagram of serum Ghrelin concentration between smokers and non-smokers.

The meta-analysis found no statistically significant difference in Ghrelin serum concentration between smokers and non-smokers (MD = 0.52[95% CI; −0.60:1.63], *p* = 0.36). Heterogeneity was observed in the studies (*I*^2^ = 68%) (Figure 8).

## Discussion

### Significance of the study

To our knowledge, this is the first comprehensive systematic review and meta-analysis investigating and summarizing the impact of smoking on leptin and ghrelin levels. This study aimed to address the current discrepancy in the literature regarding the specific impact of smoking on serum levels of leptin and ghrelin, hormones known to play a crucial role in regulating body weight with the ultimate goal of furthering our understanding of the underlying mechanisms that contribute to obesity (60).

### Summary of findings

The meta-analysis included 40 studies with a pooled total of 11,363 patients. The results showed a significant overall difference in serum leptin levels between smoking and non-smoking groups, as the serum leptin was lower in the smoking group. However, when the analysis was stratified by study design, the results varied. The case-control and cross-sectional studies showed a significant difference but not the cohort studies. Both subgroups indicated a lower leptin level in the smoking group. The subgroup difference was significant, which suggests that the difference in serum leptin levels between smoking and non-smoking groups may be influenced by the study design used. The subgrouping according to the sex revealed a significant difference in the male subgroup showing lower leptin in the smoking men compared to the non-smoking men. There was also a lower level of leptin in the smoking women group despite the non-statistically significant difference. The subgrouping according to the health condition of the population investigated revealed a significantly lower leptin level in the healthy and diabetic populations but not for pregnant and individuals with CVDs. On the other hand, no significant difference was found in the term of serum ghrelin but only six studies reported it.

### Explanation of the findings

Obesity, the relapsing, clinical syndrome, is one of the biggest worldwide health issues facing the world today (61–63). Obesity is characterized by a disrupted energy balance brought on by a high food intake in comparison to poor energy expenditure (64). Obesity is caused by a complex interplay of genetic, environmental, behavioural, and psychological variables; therefore, it is a multi-factorial condition (65). Weight is controlled by the hypothalamus’ weight-regulating centre through both long- and short-acting peripheral signals (66–69). Long-term peripheral impulses provide details regarding adipose tissue distribution. Leptin, a satiety hormone released by adipocytes, and insulin, a hormone secreted by the pancreas, are examples of these adiposity signals (70, 71). In response to eating and fasting, enteroendocrine cells emit a variety of peptide hormones, which are largely responsible for the regulation of energy balance through short-term peripheral signals (67). Gut hormones may be broadly categorized as hunger, orexigenic hormones, satiety, and anorexigenic hormones. Ghrelin is the primary known hormone secreted from the stomach in response to fasting, triggering the commencement of eating and hence controlling meal frequency (72). Obesity may result from disturbed control of gut hormone release, which may impact energy homeostasis (73). Studies found that obese individuals had a higher level of leptin (74–77) and lower ghrelin levels (77–79). Ardeshiripur et al. found that Regarding energy homeostasis and appetite control, these two ghrelin variants have different impacts. Food intake has been demonstrated to be inhibited by desacylghrelin, whereas acylghrelin is known to promote appetite and food intake. Moreover, acylghrelin has been connected to higher levels of fat and body weight, whereas desacylghrelin has been linked to lower levels of these parameters (80).

The relationship between obesity and smoking is still conflicting. Many studies showed that smokers often had lower body mass index (BMIs) than non-smokers (81–83). It was explained by the effects of nicotine on the brain, which suppresses appetite and shortens mealtimes, which leads to decreased food intake and weight loss (84, 85). According to our meta-analysis, we have established the cornerstone of the literature on smoking’s effects on leptin and ghrelin hormones and their effects on body weight.

The impact of smoking on appetite regulation is multifaceted, and it can have a variety of short- and long-term consequences on cravings and eating behaviors. While smoking is frequently associated with appetite suppression, the long-term effects can be quite different, leading to increased cravings and emotional eating. Smoking can function as an appetite reducer in the short term. Nicotine, the addictive ingredient in cigarettes, can temporarily suppress hunger and enhance metabolic rate (86, 87). This can lead to reduced food intake and possible weight loss. Many smokers claim that smoking helps them control their appetites or keep their weight in check. However, smoking’s long-term consequences on appetite regulation can be more complicated. Nicotine stimulates the brain’s reward system, resulting in the release of dopamine. a neurotransmitter associated with pleasure and reward. Through this mechanism, smoking can create conditioned responses, where the act of smoking becomes associated with pleasurable feelings (88).

The results of our study revealed that smoking lowers leptin levels, which leads to a decreased appetite, decreasing food consumption frequency and amount, and thus causing weight loss (22, 35, 71). Our finding is agreeing with the previous studies that found an inverse relationship between smoking and body BMI (84, 85). However, it is evidenced that smoking is strongly associated with increased waist circumference and abdominal obesity despite its negative effect on the body mass index (81, 82, 89–91). Leptin is known to be positively correlated with waist circumference (92, 93), presenting a complex issue that requires more research in a variety of health populations to identify the underlying factors.

The subgroup analysis by sex revealed a significant difference for males but not for females. This suggests that the effect of smoking on leptin levels may be different between men and women. This difference in the effect of smoking on leptin levels between males and females may be due to the different hormonal milieu in men and women, as well as differences in smoking behaviour and patterns (94–97). The underlying heterogeneity can be explained by the differences among the included studies in terms of the study design, differences in sample size and population characteristics. For example, the effect of smoking on hormone levels may vary among different ethnic groups or between men and women (98, 99), as discussed previously. Additionally, the degree of obesity or the presence of other health conditions may also play a role in the relationship between smoking and hormone levels.

The relationship between smoking and ghrelin levels is also mixed. Some studies have found no difference in plasma ghrelin levels between cigarette smokers and non-smokers (54, 100), while others have found that smokers had considerably greater plasma concentrations of acetylated ghrelin than non-smokers (101, 102).

The meta-analysis did not find a significant difference in ghrelin levels between smoking and non-smoking groups. The lack of significance in the meta-analysis may be due to the limited number of studies that have investigated the effect of smoking on ghrelin levels.

### Strengths

This study is a comprehensive systematic review and meta-analysis investigating the impact of smoking on leptin and ghrelin levels. The study utilized a thorough literature search and inclusion criteria to select 40 studies with a pooled total of **11,336** patients for analysis. The meta-analysis found a significant overall difference in serum leptin levels between smoking and non-smoking groups, with lower levels of leptin in the smoking group. Subgroup analysis by study design, sex, and health status also revealed significant differences. Additionally, the study is the first to investigate and summarize the impact of smoking on both leptin and ghrelin levels, providing a comprehensive overview of the topic and furthering the understanding of the underlying mechanisms that contribute to obesity.

### Limitations

This study has several limitations. Firstly, the study is based on observational studies which may be subject to bias and confounding. Secondly, the studies included in the meta-analysis were from different geographical locations, which may have led to variations in study populations and measurement methods. Thirdly, the meta-analysis is based on pooled data from multiple studies, which may have led to high heterogeneity within the subgroups and may have affected the overall results. Fourthly, the study did not take into account other factors that may affect leptin and ghrelin levels, such as diet, physical activity, and medication use, which may have led to an underestimation of the effect of smoking on these hormones. Finally, the study did not consider the duration of smoking and whether it plays a role in the relationship between smoking and hormone levels.

## Conclusion

The study included 40 studies with a pooled total of 11,336 patients, and the results showed a significant overall difference in serum leptin levels between smoking and non-smoking groups, with the smoking group having lower levels of leptin. The study also found no statistically significant difference in Ghrelin serum concentration between smokers and non-smokers.

## Data availability statement

The original contributions presented in the study are included in the article/Supplementary material, further inquiries can be directed to the corresponding author.

## Author contributions

NS: Conceptualization, Data curation, Formal analysis, Funding acquisition, Investigation, Methodology, Project administration, Resources, Software, Supervision, Validation, Visualization, Writing – original draft, Writing – review & editing. ASh: Conceptualization, Investigation, Writing – original draft, Writing – review & editing. RD: Data curation, Writing – review & editing. ASa: Data curation, Writing – review & editing. OA: Writing – original draft, Writing – review & editing. SS: Data curation, Writing – review & editing. MH: Writing – review & editing. AR: Writing – review & editing. MM: Writing – review & editing. AN: Writing – review & editing.
