# Supplementary material for: Association of serum leptin and ghrelin levels with smoking status on body weight: a systematic review and meta-analysis
Source: Front Psychiatry. 2023 Dec 4;14:1296764. doi: 10.3389/fpsyt.2023.1296764 (PMC10725976; doi:10.3389/fpsyt.2023.1296764)
Supplement: Supplementary file 1 [file Data_Sheet_1.docx]

**Supplementary 1:** Boolean keywords/search terms[**Search strategy]**

**Search strategy for PubMed:**

1. ("Smokers"[Mesh]) OR ( "Smoking"[Mesh] OR "Smoke"[Mesh] OR "Tobacco Smoke Pollution"[Mesh] OR "Smoking Water Pipes"[Mesh] OR "Non-Smokers"[Mesh] OR "Smoke-Free Policy"[Mesh] OR "Smoking, Non-Tobacco Products"[Mesh] OR "Smoking Cessation"[Mesh] OR "Marijuana Smoking"[Mesh] OR "Pipe Smoking"[Mesh] OR "Smoking Prevention"[Mesh] OR "Smoking Pipes"[Mesh] OR "Tobacco Smoking"[Mesh] OR "Water Pipe Smoking"[Mesh] OR "Cigarette Smoking"[Mesh] OR "Cigar Smoking"[Mesh] OR "Smoke Inhalation Injury"[Mesh] )*)

**OR**

1. ("Nicotine"[Mesh]) OR (“Electronic Nicotine Delivery Systems"[Mesh] OR "Nicotine Chewing Gum"[Mesh] OR "Tobacco Use Cessation Devices"[Mesh] OR “nicotine 1-N-oxide" [Supplementary Concept])

**AND**

1. ("Leptin"[Mesh]) OR (“Receptors, Leptin"[Mesh] OR "leptin, Ala (39,40,41)-" [Supplementary Concept] OR “leptin (116-130) amide" [Supplementary Concept])

**OR**

1. ("Ghrelin"[Mesh]) OR (“Receptors, Ghrelin"[Mesh] OR "ghrelin, des-n-octanoyl" [Supplementary Concept] OR “ghrelin, des-Gln (14)-" [Supplementary Concept])

**Search strategy for Scopus:**

1. (TITLE-ABS-KEY(Smokers OR Smoking OR Smoke OR Tobacco Smoke Pollution OR Smoking Water Pipes OR Non-Smokers OR Smoke-Free Policy OR Smoking, Non-Tobacco Products OR Smoking Cessation OR Marijuana Smoking OR Pipe Smoking OR Smoking Prevention OR Smoking Pipes OR Tobacco Smoking OR Water Pipe Smoking OR Cigarette Smoking OR Cigar Smoking OR Smoke Inhalation Injury))
2. OR (TITLE-ABS-KEY(Nicotine OR Electronic Nicotine Delivery Systems OR Nicotine Chewing Gum OR Tobacco Use Cessation Devices OR nicotine 1-N-oxide))
3. OR (TITLE-ABS-KEY(Leptin OR Receptors, Leptin OR leptin, Ala (39,40,41)- OR leptin (116-130) amide))
4. OR (TITLE-ABS-KEY(Ghrelin OR Receptors, Ghrelin OR ghrelin, des-n-octanoyl OR ghrelin, des-Gln (14)-))
5. LIMIT-TO (LANGUAGE, "English")
6. LIMIT-TO (DT, "within 5 years")
7. LIMIT-TO (DOCTYPE, "ar" OR "cp" OR "re")
8. LIMIT-TO (SUBJAREA, "PUBLIC, ENVIRONMENTAL & OCCUPATIONAL HEALTH" OR "ENDOCRINOLOGY & METABOLISM" OR "NUTRITION & DIETETICS")

**Search strategy for Web of Science:**

1. (("Smokers"[Mesh]) OR ("Smoking"[Mesh]) OR "Smoke"[Mesh] OR "Tobacco Smoke Pollution"[Mesh] OR "Smoking Water Pipes"[Mesh] OR "Non-Smokers"[Mesh] OR "Smoke-Free Policy"[Mesh] OR "Smoking, Non-Tobacco Products"[Mesh] OR "Smoking Cessation"[Mesh] OR "Marijuana Smoking"[Mesh] OR "Pipe Smoking"[Mesh] OR "Smoking Prevention"[Mesh] OR "Smoking Pipes"[Mesh] OR "Tobacco Smoking"[Mesh] OR "Water Pipe Smoking"[Mesh] OR "Cigarette Smoking"[Mesh] OR "Cigar Smoking"[Mesh] OR "Smoke Inhalation Injury"[Mesh])
2. ("Nicotine"[Mesh]) OR ("Electronic Nicotine Delivery Systems"[Mesh] OR "Nicotine Chewing Gum"[Mesh] OR "Tobacco Use Cessation Devices"[Mesh])
3. ("Leptin"[Mesh]) OR ("Receptors, Leptin"[Mesh])
4. ("Ghrelin"[Mesh]) OR ("Receptors, Ghrelin"[Mesh])

**Supplementary 2:**

**Quality assessment:**

| CASE CONTROL STUDIES | | | | | | | | | |
| --- | --- | --- | --- | --- | --- | --- | --- | --- | --- |
| Study ID | Selection | | | | Comparability | Exposure | | | Quality score |
|  | Is the case definition adequate? | Representativeness of  the cases | Selection of Controls | Definition of  Controls | Comparability of cases and controls on the basis of the design or analysis | Ascertainment of  exposure | Same method of ascertainment for cases and controls | Non-Response  rate |  |
| Bokarewa 2014 | * | * |  |  | ** | * | * |  | Fair |
| Ozkan 2005 | * | * | * | * | * | * | * |  | Good |
| Der Goltz 2010 | * | * | * | * | * | * | * | * | Good |
| Klein 2004 | * |  | * |  | * | * | * |  | Fair |
| Perkins 2002 | * | * | * | * | * | * | * |  | Good |
| Bergmann 2009 |  | * |  | * |  | * |  |  | Poor |
| Nicklas BJ,1999 | * | * | * | * | * | * | * |  | Good |
| Hakan Ekmekci,2005 | * |  |  | * | ** |  | * |  | Poor |
| Bayram Koc,2009 | * |  | * | * | * | * | * |  | Good |
| Sana S. Al Mutairi,2008 | * |  | * | * | ** | * | * | * | Good |
| Oliver Pérez-Bautista 2018 | * | * | * | * | * | * | * |  | Good |
| Natacha Bouhours-Nouet 2006 | * | * | * | * | * | * | * |  | Good |
| Ki-Woong Kim, 2014 | * | * | * | * | * |  |  | * | Poor |
| SWATHI S RAO,2020 | * | * | * | * | * |  |  |  | Poor |
| Kadir Aşçibaşi, 2018 | * |  | * | * | * | * | * | * | Good |
| Xiao-Jun Bai, 2015 | * | * | * | * | * | * | * |  | Good |
| Tajamul Hussain, 2012 | * | * | * | * | ** | * | * |  | Good |

| **Cross-Sectional Studies** | | | | | | | | |
| --- | --- | --- | --- | --- | --- | --- | --- | --- |
| **Study ID** | **Selection** | | | | **Comparability** | **Outcome** | | **Quality  Score** |
|  | **Representativeness of the sample** | **Sample size** | **Non-respondents** | **Ascertainment of the exposure (risk factor)** | **The subjects in different outcome groups are comparable, based on the study design or analysis. Confounding factors are controlled.** | **Assessment of the outcome** | **Statistical test** |  |
| Kianoush 2018 | * |  |  | * | * |  | * | Poor |
| Silva Gomes 2015 | * |  |  |  | ** | ** | * | Poor |
| Shintaro Nagayasu, 2012 |  |  | * |  | * | * | * | Poor |
| Mustafa al'Absi ,2010 | * |  | * | * | ** |  | * | Poor |
| Nazan Cobanoglu, 2012 | * | * | * | * | ** | ** | * | Good |
| M. Hara, M. Togo 2001 | * | * |  | * | * | * |  | Poor |
| H. Larsson 2001 | * |  |  | * | * | * | * | Fair |
| MOTOTAKA YOSHINARI 1998 | * | * |  |  | ** | * | * | Fair |
| Nasser 2008 |  |  |  | * | * | * | * | Poor |
| MING WEI,1997 | * | * |  | * | * | * |  | Poor |
| REGIS COUTANT,2001 | * | * |  | * | * | * | * | Good |
| Lucas 2013 | * | * |  | * |  | * | * | Poor |
| Machado, 2021 | * | * |  |  | ** | * | * | Fair |
| Yahia A. Kaabi, 2014 |  |  |  | * | * | * | * | Poor |
| Targher, 2001 | * |  |  | * |  | * |  | Poor |

| **COHORT STUDIES** | | | | | | | | | |
| --- | --- | --- | --- | --- | --- | --- | --- | --- | --- |
| **Study ID** | **Selection** | | | | **Comparability** | **Outcome** | | | **Quality Score** |
|  | **Representativeness of the exposed cohort** | **Selection of the non-exposed cohort** | **Ascertainment of exposure** | **Demonstration that outcome of interest was not present at start of study** | **Comparability of cohorts on the basis of the design or analysis** | **Assessment of outcome** | **Was follow-up long enough for outcomes to occur** | **Adequacy of follow up of cohorts** |  |
| Hilawe 2014 | * | * | * |  | * | * | * | * | Good |
| B Helland,2001 | * | * | * | * | ** | * | * | * | Good |
| Lisa J. Martin, 2002 | * | * |  |  | * | * |  | * | Fair |
| Maria Kryfti, 2015 | * | * | * |  | * | * | * | * | Good |
| Heejin Lee ,2006 |  | * |  |  | * |  | * | * | Poor |
| Christos S. Mantzoros 2000 | * | * | * | * |  | * | * | * | Good |
| Marietta Stadler, 2014 |  | * | * |  |  | * | * |  | Fair |
| Mutschler, 2012 |  |  | * | * |  | * |  | * | Poor |
